# A Role for Polyploidy in the Tumorigenicity of Pim-1-Expressing Human Prostate and Mammary Epithelial Cells

**DOI:** 10.1371/journal.pone.0002572

**Published:** 2008-07-02

**Authors:** Meejeon Roh, Omar E. Franco, Simon W. Hayward, Riet van der Meer, Sarki A. Abdulkadir

**Affiliations:** 1 Department of Pathology, Vanderbilt University Medical Center, Nashville, Tennessee, United States of America; 2 Department of Urology, Vanderbilt University Medical Center, Nashville, Tennessee, United States of America; University of Minnesota, United States of America

## Abstract

**Background:**

Polyploidy is a prominent feature of many human cancers, and it has long been hypothesized that polyploidy may contribute to tumorigenesis by promoting genomic instability. In this study, we investigated whether polyploidy *per se* induced by a relevant oncogene can promote genomic instability and tumorigenicity in human epithelial cells.

**Principal Findings:**

When the oncogenic serine-threonine kinase Pim-1 is overexpressed in immortalized, non-tumorigenic human prostate and mammary epithelial cells, these cells gradually converted to polyploidy and became tumorigenic. To assess the contribution of polyploidy to tumorigenicity, we obtained sorted, matched populations of diploid and polyploid cells expressing equivalent levels of the Pim-1 protein. Spectral karyotyping revealed evidence of emerging numerical and structural chromosomal abnormalities in polyploid cells, supporting the proposition that polyploidy promotes chromosomal instability. Polyploid cells displayed an intact p53/p21 pathway, indicating that the viability of polyploid cells in this system is not dependent on the inactivation of the p53 signaling pathway. Remarkably, only the sorted polyploid cells were tumorigenic *in vitro* and *in vivo*.

**Conclusions:**

Our results support the notion that polyploidy can promote chromosomal instability and the initiation of tumorigenesis in human epithelial cells.

## Introduction

Aneuploidy is a common characteristic of human cancers and has been proposed as a driver of tumorigenesis [1], [2]. During tumor initiation, aneuploidy may arise via polyploidization where unstable tetraploid intermediates cause further chromosomal abnormalities including chromosomal gains, losses and translocations [3], [4]. In human tumors, aneuploidy is found in pre-cancerous lesions of the cervix [5]–[7], head and neck [8], colon [5], [9], esophagus [10] and prostate [11]. Nevertheless, the question of whether genomic instability is a driving force for cancer development, or a consequence of tumorigenesis has remained the subject of debate [12]. A recent study by Fujiwara *et al* has provided experimental support for a role for polyploidy in tumorigenesis by using p53-null tetraploid mouse mammary epithelial cells [13]. These authors showed that tetraploid cells generated by transient treatment with a cytokinesis inhibitor, dihydrocytochalasin B, were tumorigenic in vivo. However, the impact of aneuploidy on tumorigenicity can be context dependent. In mice with reduced levels of the mitosis-specific, centromere-linked motor protein CENP-E, the resulting aneuploidy promotes tumorigenicity in some tissues while suppressing tumor development in others [14]. These findings underscore the importance of assessing the roles of chromosomal instability in tumorigenesis in specific cell types and animal species.

In the present study we have examined the question of whether polyploidy can promote tumorigenesis in human epithelial cells using a model of spontaneous polyploidy induced by the oncogene Pim-1. The Pim-1 oncogene is a serine-threonine kinase implicated in the development of various tumors including lymphomas and prostate carcinomas [15]–[17]. We have previously demonstrated that overexpression of Pim-1 in human prostate and breast epithelial cells results in the gradual emergence of polyploidy [18], [19]. Notably, Pim-1 is abundantly expressed in the megakaryocyte lineage where it is involved in the regulation of polyploidy [20], suggesting that Pim-1 induced polyploidy in tumorigenesis might be a pathological manifestation of the same process. As the evolution of polyploidy in Pim-1 expressing cells is stochastic [19], this allowed us to obtained sorted Pim-1 expressing cells of the same passage that are either diploid or polyploid based on their DNA content. Our studies using these cells indicate that polyploidy induced by Pim-1 can promote the development of chromosomal abnormalities and tumorigenicity in human prostate and mammary epithelial cells.

## Results

### Polyploid Pim-1-expressing RWPE1 Prostate Cells are Tumorigenic *in Vivo*

**Figure 1:**
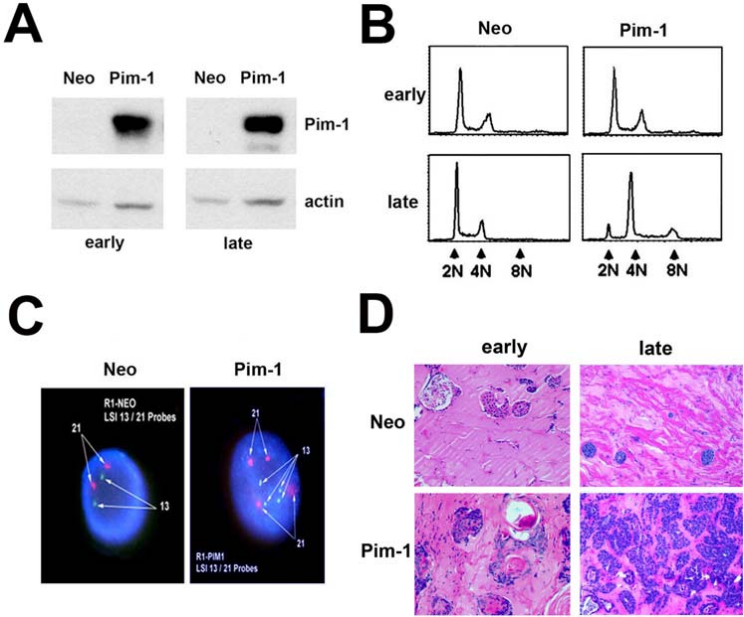
Late passage, polyploid, Pim-1-expressing RWPE1 prostate cells are tumorigenic in nude mice. (A) Western blotting for Pim-1 and actin in RWPE1 cells overexpressing Pim-1 or vector control (Neo). Early and late passage cells are shown. (B) Cell cycle profiles of Pim-1 overexpressing RWPE1 cells shows polyploidy in late passage cells. (C) FISH analysis of late passage Pim-1 overexpressing RWPE1 cells using probes for chromosomes 13 and 21 show chromosome doubling. (D) Sample images of H&E stained sections from RWPE1 xenografts. Only late passage Pim-1 expressing cells were tumorigenic.

To examine the oncogenic functions of Pim-1 in prostate epithelial cells, we stably overexpressed Pim-1 in immortalized, non-tumorigenic prostate epithelial RWPE1 cells (Figure 1A). As reported previously [18], [19], late passage RWPE1-Pim-1 cells are polyploid (tetraploid) as shown by FACS for DNA content and FISH, while early passage cells are diploid (Figures 1B, C). We injected these cells with matrigel into the flanks of nude mice subcutaneously to form xenografts. Analysis of the xenograft tissue indicates that only late passage RWPE1-Pim-1 cells formed small tumors (40% incidence; n = 10, average tumor volume = 44.02±12.03 mm^3^ at time of sacrifice) while both early and late passage control RWPE1-Neo cells (n = 10 each) as well as early passage RWPE1-Pim-1 cells (n = 10) only formed small benign looking glands (Figure 1D). Thus in this assay, tumorigenicity appears to depend on both Pim-1 expression and prolonged passage and/or polyploidy.

### Isolation of Matched Diploid/Polyploid Pim-1-expressing RWPE1 Cells by Cell Sorting

**Figure 2:**
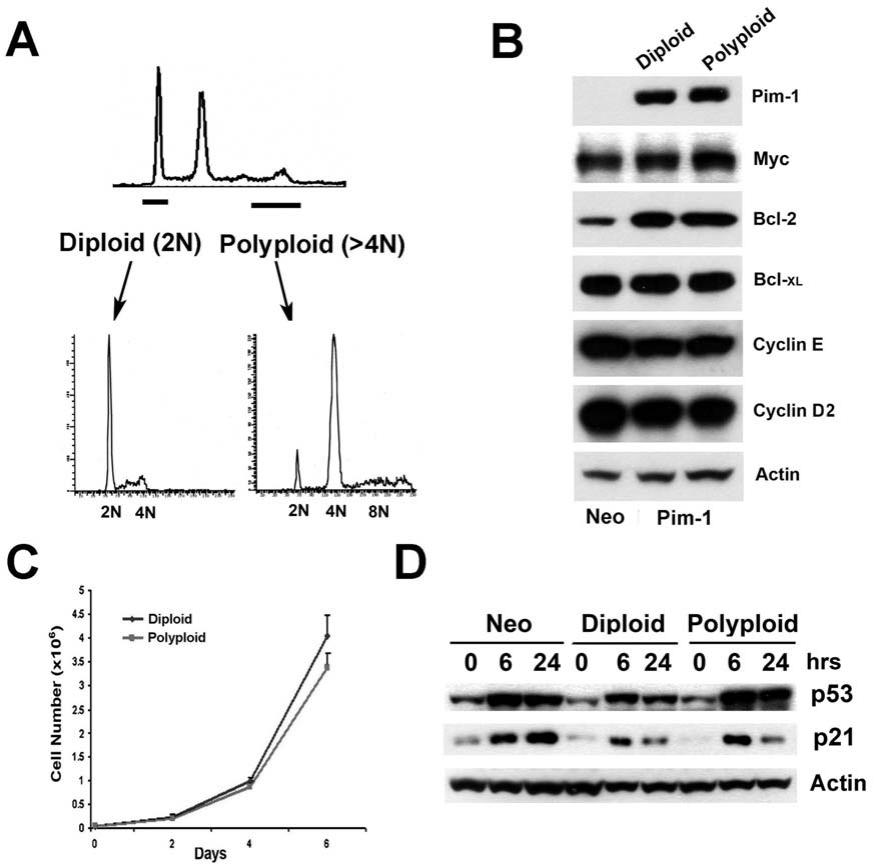
Isolation of matched diploid/polyploid RWPE1-Pim-1 cells by cell sorting. (A) Diagram showing scheme for isolation of diploid (2N) and polyploid (>4N) Pim-1 cells by FACS sorting based on DNA content. Bottom panel is the FACS profile of sorted cells after several passages to get enough cells for FACS analysis. (B) Western blotting of Pim-1 and other markers in FACS sorted cells shows similar expression levels in sorted diploid and polyploid cells. (C) Cell counting of diploid and polyploid Pim-1 overexpressing cells. (D) Western blotting shows that the p53 pathway is intact in all FACS-sorted RWPE1 cells as demonstrated by the induction of p53 and p21 following daunorubicin treatment.

The results of our xenograft experiments suggested that polyploidy might have been a contributing factor in the tumorigenicity of late passage RWPE1-Pim-1 cells, since control non-polyploid RWPE1-Neo cells of the same late passage as well as early passage Pim-1-expressing cells were not tumorigenic. To directly investigate this, we took advantage of the gradual nature by which polyploidy arises in cultures of Pim-1-expressing cells. We have previously shown, using three different experimental approaches that all Pim-1-expressing cells have the potential to become polyploid, and do so in a stochastic manner [19]. We sorted intermediate passage cells based on DNA content by FACS to obtain matched diploid (2N) and polyploid (≥4N) cell populations of the same passage (Figure 2A). After sorting, the cells stably maintained a diploid or tetraploid profile by FACS. Importantly, the expression levels of Pim-1 as well as those of several cell cycle and anti-apoptotic molecules (including Myc, Cyclin E, Cyclin D2, Bcl-2 and Bcl-_XL_) were similar in both diploid and polyploid cells (Figure 2B). Notably, Bcl-2, which is a known target of Pim-1 [21], is upregulated in RWPE1-Pim-1 cells relative to control RWPE1-Neo cells; however the sorted RWPE1-Pim-1 diploid and polyploid cells expressed equivalent levels of Bcl-2 (Figure 2B). We found no significant differences in the proliferation rates of diploid and polyploid RWPE1-Pim-1 cells *in vitro* (Figure 2C).

Previous studies have suggested the existence of a p53-dependent checkpoint –the “tetraploidy checkpoint”- that limits the proliferation of polyploid cells, although the existence of such a “tetraploidy checkpoint” has been contested [4], [22]. To determine if the p53 signaling pathway is inactivated in the sorted polyploid cells, we treated the cells with the chemotherapeutic agent daunorubicin. We observed stabilization of p53 as well as the induction of its target molecule, p21 after daunorubicin treatment, indicating that the p53 pathway is intact in these cells (Figure 2D). This may appear surprising since RWPE1 cells were immortalized by human papillomavirus type 18 (HPV-18) and the E6 protein of HPV-18 is known to interfere with p53 function [23]. Nevertheless, it has been reported that the p53 pathway is functional in certain HPV-immortalized cell lines [24], consistent with our results.

### Chromosomal Abnormalities in Polyploid RWPE1-Pim-1 Cells

**Figure 3:**
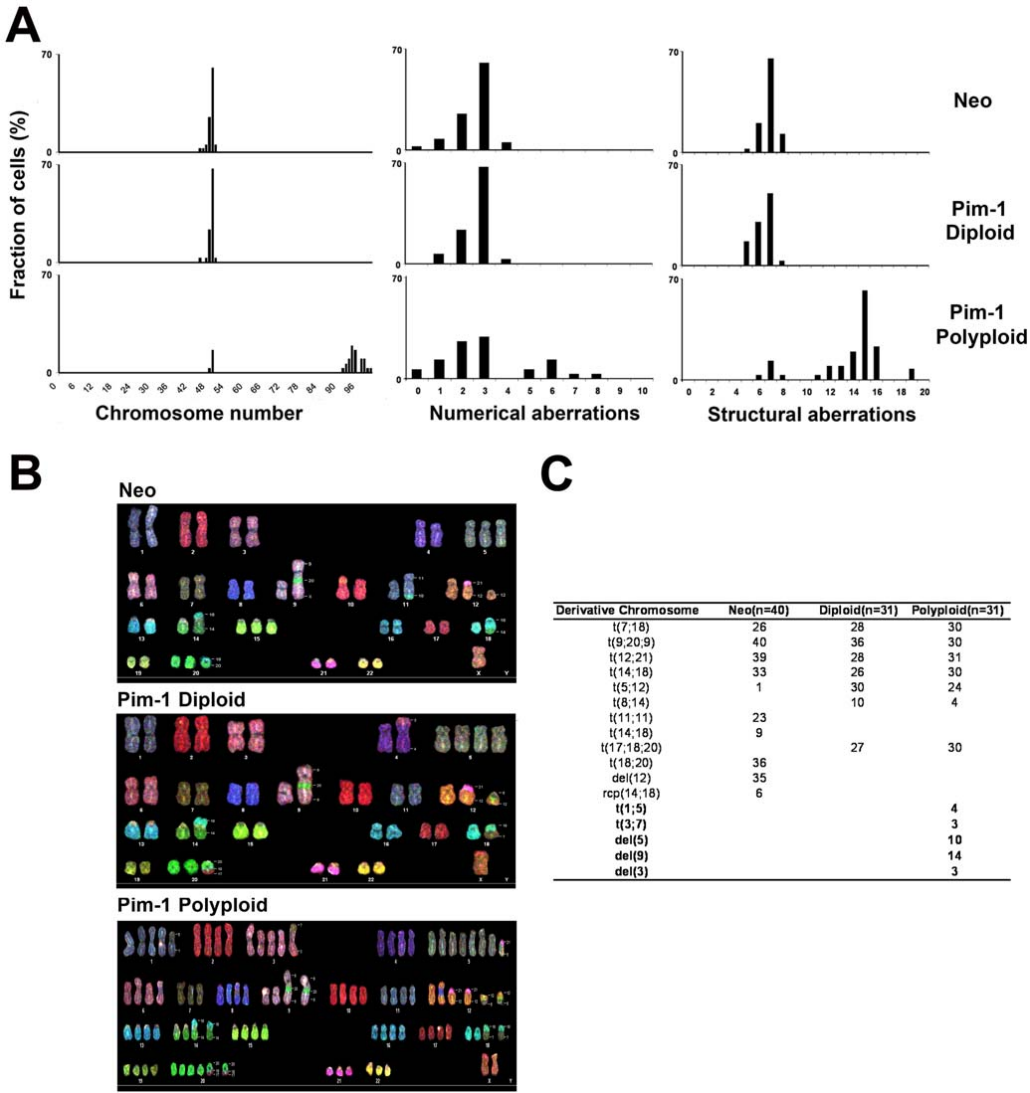
Chromosomal abnormalities in sorted polyploid RWPE1-Pim-1 cells. (A) Percentage of cells showing total chromosome number, numerical and structural aberrations from FACS-sorted RWPE1 cells. Total 31 to 40 metaphase spreads were analyzed per sample and scored for the chromosome number, numerical aberrations, and structural aberrations. (B) Representative SKY images of sorted Neo, diploid and polyploid RWPE1-Pim-1 cells. Neo: 49,X,del(Y),+5, t(9;20;X),t(11;18),+12,del(12),t(12;21),t(14;18),+15,t(14;18),+20,t(18;20). Diploid: 49,X,del(Y),t(3;4),+5,+5,t(9;20;9),+12,t(5;12)(12;21),t(14;18),t(7;18),+20,t(17;18;20). Polyploid: 97,XX,del(Y),+1,+1,+1,t(1;5),+2,+2,+3,+3,+3,t(3;7),+4,+4,+5,+5,+5,+5,+5,+5,t(5;21),+6,+6,+7,+8,+8,+9,+9,t(9;20;9)×2,+10,+10,+11,+11,+12,+12,+12,+12,t(5;12)×2(12;21)×2,+13,+13,+14,+14,t(14;18)×2,+15,+15,+16,+16,+17,+17,+18,+18,t(7;18)×2,+19,+19,+20,+20,+20,+20,t(17;18;20)×2,+21,+22. (C) Table showing structural chromosomal aberrations. The numbers of derivative chromosomes observed are shown. Polyploid-specific abnormalities are shown in bold.

To gain further insight into the genomic alterations in polyploid cells, we performed karyotype analyses. Spectral karyotyping (SKY) showed that 100% of the control RWPE1-Neo (n = 40) and diploid RWPE1-Pim-1 cells (n = 31) cells examined were near-diploid, containing between 45 and 50 chromosomes per cell (Figures 3A, B). By contrast, a majority of the polyploid RWPE1-Pim-1 cells (81%, n = 31) were near-tetraploid, containing 91–100 chromosomes. Apart from whole-chromosomal gains, Pim-1 polyploid cells exhibited a wide range of both numerical and structural aberrations including chromosomal translocations and deletions (Figures 3B, C), whereas control Neo and Pim-1 diploid cells had fewer structural abnormalities and numerical variations. Although the number of structural chromosomal aberrations per cell in polyploid cells was approximately twice that in control Neo or diploid cells, the number of structural chromosomal aberrations per chromosome was almost the same in all groups (Figure S1), suggesting that the higher chromosome number in polyploid cells is a primary factor in the increase in chromosomal abnormalities observed. Nevertheless, although a majority of polyploid cells shared the same chromosomal translocation and deletion with control Neo and diploid cells, attesting to their common origin, there were also several polyploid cell-specific chromosomal translocations and deletions present in various fractions of the polyploid cells (Figure 3C). These results indicate the presence of chromosomal instability and the emergence of aneuploidy in the sorted polyploid cells.

### Sorted, Polyploid RWPE1 Prostate Cells are Tumorigenic *in Vitro* and *in Vivo*

**Figure 4:**
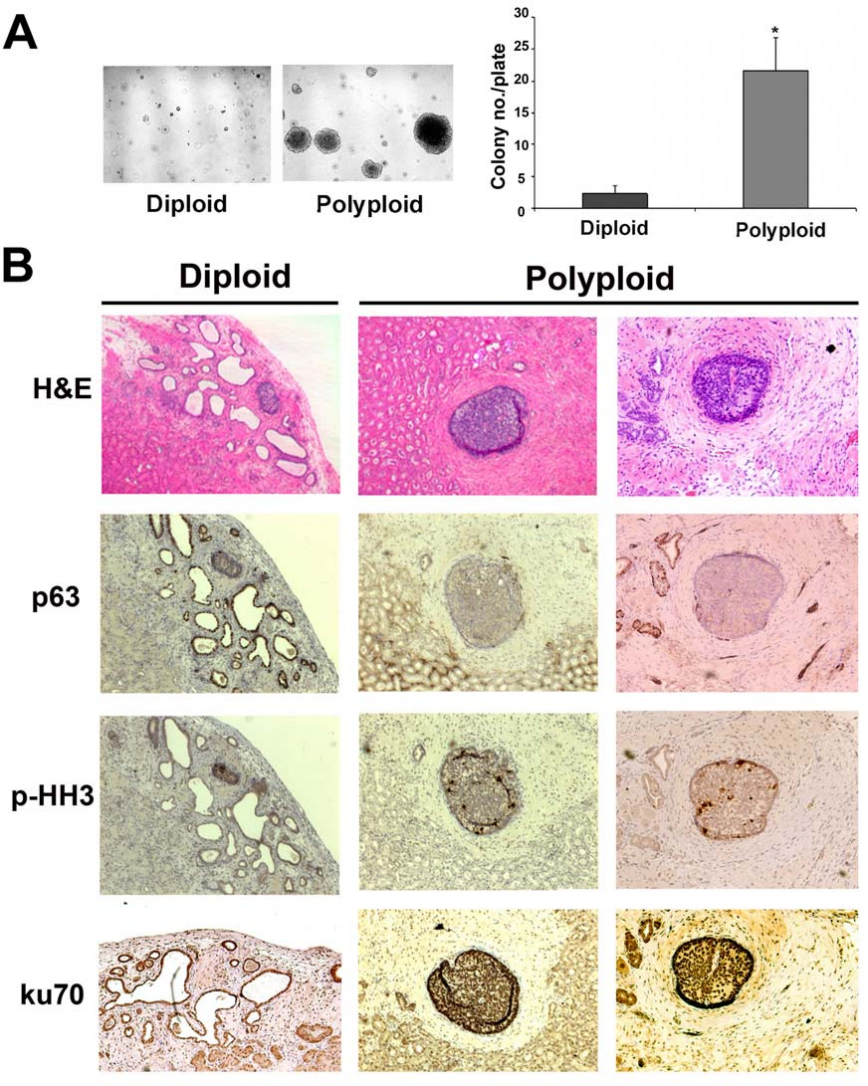
Polyploidy promotes the tumorigenicity of RWPE1 prostate cells *in vitro* and *in vivo*. (A) Soft agar assay with colony counting in FACS-sorted RWPE1 cells. Colonies larger than 0.5 mm in diameter were counted. Results represent average of triplicate experiments. *p<0.05. (B) Histological analysis of grafts of diploid and polyploid RWPE1-Pim-1 cells recombined with rat UGM and grafted under the kidney capsule. Grafts from polyploid cells contained foci of carcinoma-in-situ with loss of the basal cell marker p63 and high rates of mitotic cells (p-HH3, phospho-histone H3) while diploid cells formed largely small benign looking glands that express p63 and low rates of mitosis (p-HH3). A human-specific Ku70 antibody was used to confirm the human origin of glands. All images were taken at the same magnification of 4×.

We next examined the role of polyploidy and the ensuing chromosomal instability in the *in vitr*o tumorigenicity of RWPE1-Pim-1 cells by assessing the ability of sorted cells to grow in an anchorage-independent manner in soft agar. As shown in Figure 4A, only polyploid RWPE1-Pim-1 cells formed colonies in soft agar, despite the fact that the expression levels of Pim-1 are similar in both diploid and polyploid cells as noted earlier in Figure 2B and these cells were carried for the same passage. These data indicate that Pim-1 expression alone is insufficient to promote growth in soft agar and that polyploidy (and the resultant genomic instability) can cooperate with Pim-1 to promote the tumorigenicity of prostate epithelial cells. To determine if genomic instability due to polyploidy can promote tumorigenesis *in vivo*, we performed tissue recombination experiments. Human prostate epithelium is known to have the ability to generate prostate gland-like structures when combined with rat urogenital mesenchyme (UGM) and implanted under the renal capsule of immune-deficient mice [25]. We combined the sorted diploid and polyploid RWPE1-Pim-1 as well as control RWPE1-Neo cells with UGM and grafted them under the renal capsule of immune-deficient mice.

Twelve weeks after grafting, all of the grafts from control Neo (n = 4) and diploid cells (n = 8) formed largely normal looking, benign gland structures. However, 3 of 8 grafts from polyploid cells contained foci of carcinoma in situ (Figure 4B) with evidence of loss of the basal cell marker p63 and increased levels of mitosis as shown by staining for phospho-histone H3 (Figure 4B). To confirm the RWPE1 origin of the glands, human-specific Ku70 staining was used. Taken together, these results indicate that polyploidy induced by Pim-1 promotes genomic instability which contributes to tumorigenicity.

### Polyploid, Pim-1-expressing Telomerase-Immortalized Mammary Epithelial Cells are Tumorigenic

**Figure 5:**
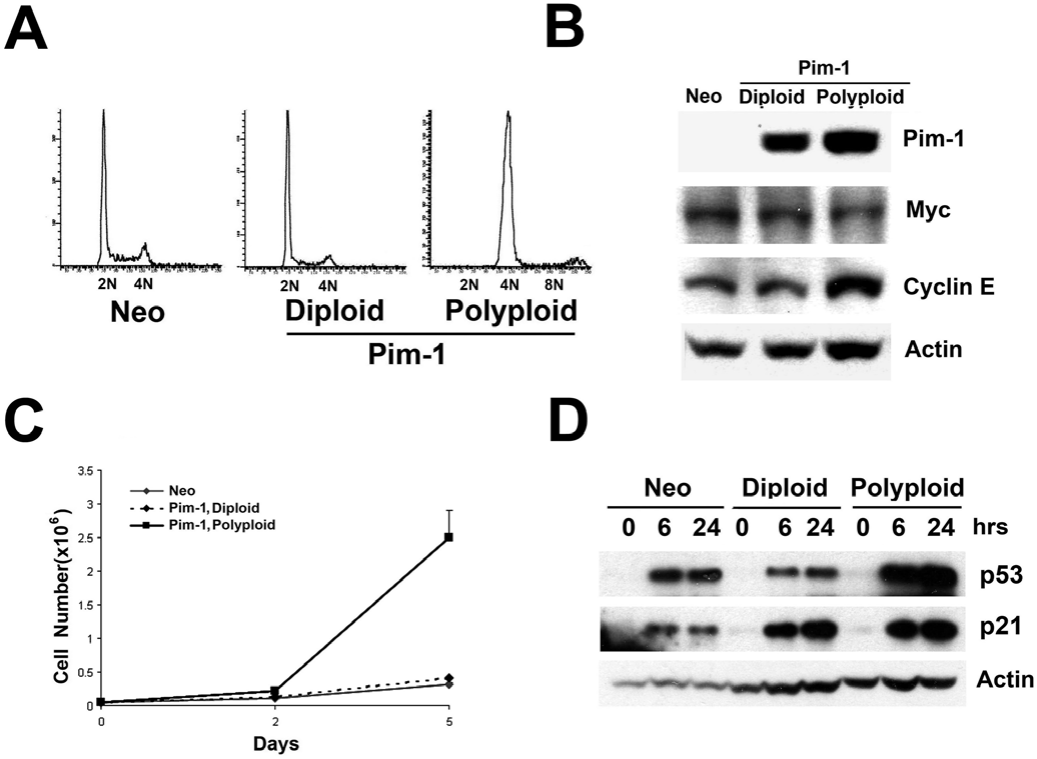
FACS sorted, Polyploid, Pim-1 expressing telomerase-immortalized mammary epithelial cells (hTERT-HME1) are tumorigenic *in vitro*. (A) FACS profile after cell sorting. hTERT-HME1 cells were sorted based on DNA content. (B) Western blotting of Pim-1 and other markers in sorted cells. Pim-1 expression levels are similar in diploid and polyploid cells. (C) Cell counting of Neo control, diploid and polyploid Pim-1 overexpressing cells. (D) Western blotting for p21 and p53 after daunorubicin treatment shows that p53 function is intact in all FACS-sorted hTERT-HME1 cells.

We next sought to determine if the role of Pim-1-induced polyploidy in promoting genomic instability and tumorigenicity extends to other human cell types. In addition, we wanted to examine this phenomenon in cells immortalized through means other than an oncogenic virus (i.e. HPV). We used hTERT-HME1 cells which are non-tumorigenic human mammary epithelial cells immortalized by expression of the human telomerase catalytic subunit, hTERT. Similar to our findings with the RWPE1 prostate cells, we observed that only polyploid, late passage Pim-1 expressing hTERT-HME1 cells formed colonies in soft agar (Figure S2). Next, we isolated matched diploid/polyploid Pim-1-expressing hTERT-HME1 cells of the same passage by cell sorting. The FACS profile of sorted cells showed that Pim-1 diploid and control Neo derived-cells maintained diploid DNA content, whereas Pim-1 polyploid cells were almost exclusively tetraploid (Figure 5A). The expression levels of Pim-1 as well as Myc were comparable in both the diploid and polyploid hTERT-HME1 cells (Figure 5B). We observed a modest increase in Cyclin E in the polyploid cells, which may be related to the increased proliferation rate of the hTERT-HME1-derived polyploid cells *in vitro* (Figure 5C). This contrasts with the case for RWPE1-derived polyploid cells which displayed comparable proliferation rate relative to their diploid counterparts. Furthermore, we examined the expression of various cell markers in polyploid cells including early progenitor cell markers CD44, nestin and cytokeratin 5 by immunofluorescence. We found no significant differences between diploid and polyploid cells (Figure S3). We also probed the p53 pathway in hTERT-HME1-derived polyploid cells by treating the cells with daunorubicin followed by western blotting for p53 and p21. The results indicate that as in the polyploid RWPE1-derived cells this pathway is intact in the hTERT-HME1 cells (Figure 5D). Thus the viability of the polyploid cells examined here does not appear to depend on the inactivation of p53. Since Pim-1 has pro-survival functions, it may substitute for p53 loss in promoting the viability of emerging polyploid cells.

### Chromosomal Abnormalities in Polyploid hTERT-HME1 Cells

**Figure 6:**
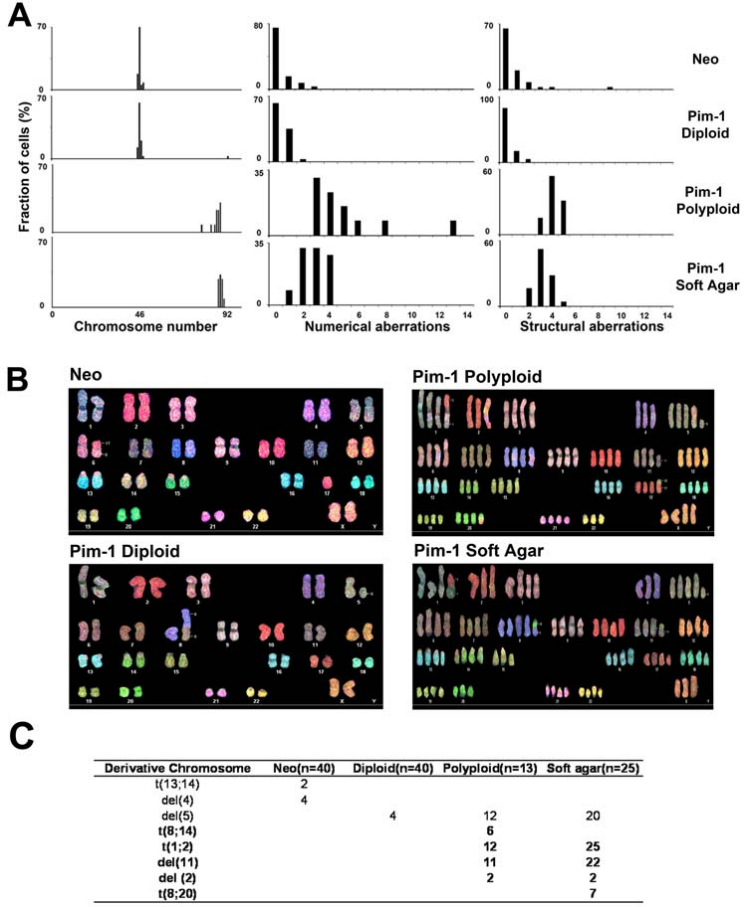
Chromosomal abnormalities in polyploid hTERT-HME1 cells. (A) Percentage of cells showing total chromosome number, numerical and structural aberrations from FACS-sorted hTERT-HME1 cells. Note that karyotypes of polyploid and soft agar derived cells are highly heterogeneous, whereas karyotypes of control Neo and diploid cells are more uniform. Total 13 to 40 metaphase spreads were analyzed per sample and scored for the chromosome number, numerical aberrations, and structural aberrations. (B) Representative SKY images from each FACS-sorted hTERT-HME1 cells. Neo: 45,XX,t(6;17),−17. Diploid: 46,XX,del(5),t(5;8). Polyploid: 89,XXXX,+1,+1,t(1;2),+2,+3,+3,+4,+5,+5,+5,del(5),+6,+6,+7,+7,+8,+8,+9,+9,+10,+10,+11,+11,del(11),+12,+12,+13,+13,+14,+15,+15,+16,+16,+17,+17,t(15;17),+18,+18,+19,+19,+20,+20,+21,+21,+22. Soft agar: 89,XXX,+1,+1,t(1;2),+2,+3,+3,+4,+5,+5,+5,del(5), +6,+6,+7,+7,+8,+8,+8,t(8;20),+9,+9,+10,+10,+11,+11,del(11),+12,+12,+13,+13,+14,+14,+15,+16,+16,+17,+17,+18,+18,+19,+19,+20,+21,+21,+22,+22. (C) Tables showing derivative chromosomes in the different sorted cell types. The numbers of derivative chromosomes observed are shown. Polyploid and soft agar derived cell-specific abnormalities are shown in bold.

**Figure 7:**
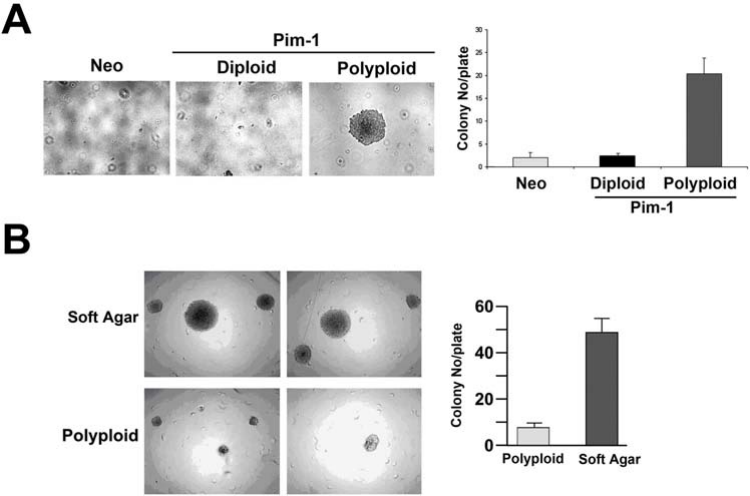
Polyploidy promotes the tumorigenicity of hTERT-HME1 cells. Soft agar assay of FACS-sorted diploid and polyploid hTERT-HME1 Pim-1 overexpressing cells and control Neo cells. Note the larger colonies in polyploid cells. Colonies larger than 1 mm in diameter were counted from triplicate of 60 mm dishes. Original magnification: 10×. (B) We isolated cells from soft agar colonies formed by polyploid cells shown in Fig. 7A. The tumorigenic potential of these soft agar-derived polyploid cells was compared side by side with that of the parental polyploid cells by soft agar assay. The soft agar-derived cells formed larger size colonies with higher frequency than the parental polyploid cell population. Original magnification; 4×.

The karyotypes of control Neo and diploid Pim-1-hTERT-HME1 cells were stable and uniform with few numerical and structural abnormalities (Figure 6). All of the Neo control cells examined (n = 40) and almost all of the diploid Pim-1 cells (39 of 40) were diploid or near-diploid, containing between 45 and 48 chromosomes. In contrast, all polyploid cells (n = 13) contained 79 to 89 chromosomal numbers, indicative of near-tetraploidy (Figure 6A). The observed chromosome numbers in the polyploid cells (79–89 chromosomes) are lower than the predicted doubling of the number of chromosomes in the Neo/Diploid cells (90–98 chromosomes). This suggests that sub-tetraploid cell population could be derived either from tetraploid cells by ongoing chromosomal loss, or from the diploid population by unknown mechanisms. Furthermore, the polyploid cells displayed significantly more structural chromosomal abnormalities than the control cells (Figures 6A–C). To gain additional insights into the evolution of tumorigenic subpopulations among polyploid cells, we isolated and cultured cells from soft agar colonies derived from polyploid cells [see Figure 7A below]. By SKY analysis, soft agar-derived cells displayed increased numerical and structural aberrations similar to the polyploid cells (Figures 6A–C). In addition, the numbers of structural chromosomal aberrations per chromosome were higher in the polyploid and soft agar-derived cells compared to the control Neo or diploid cells (Figure S1). These results suggest that the chromosomal abnormalities seen in these cells are not merely due to increased chromosomal numbers but are due to active chromosomal instability. Notably, 7 out of 25 of the soft agar-derived cells examined contained a unique chromosomal translocation, t(8;20), providing further evidence of chromosomal instability in the evolution of tumorigenic populations (Figure 6C).

### Sorted, Polyploid hTERT-HME1 Mammary Cells are Tumorigenic

The tumorigenicity of the sorted hTERT-HME1 cells was tested by anchorage-independent growth in soft agar. Polyploid cells formed robust colonies in soft agar (Figure 7A), unlike the diploid or Neo control cells, indicating that the pro-tumorigenic effect of Pim-1-induced polyploidy extends to different cell types. Furthermore, the soft agar derived cells formed larger sized colonies with higher frequency than polyploid cells, suggesting more tumorigenic potential of these cells (Figure 7B).

## Discussion

In this study we demonstrated that polyploidy, induced by the oncogenic kinase Pim-1 can promote the development of aneuploidy and tumorigenicity in human epithelial cells. Using genetically matched FACS-sorted diploid and polyploid prostate and mammary epithelial cells, we demonstrated that only the polyploid cells were tumorigenic despite the fact that they expressed equivalent levels of the Pim-1 oncogene. Thus, Pim-1 overexpression itself in the absence of polyploidy is not sufficient for tumorigenicity in our experimental system. We also provide evidence that the tumorigenicity of polyploid cells is likely due to the intrinsic chromosomal instability present in these cells including whole-chromosome gains/losses as well as structural abnormalities including deletions and translocation. Furthermore, our data demonstrated that the survival of polyploid cells is not dependent on inactivation of the p53 pathway, suggesting that Pim-1 might substitute for p53 loss.

Genomic instability is a hallmark of human cancer and a majority of carcinomas display gross chromosomal abnormalities. It is thought that polyploidization (specifically tetraploidy) may represent an important intermediate step in tumor initiation via its ability to catalyze the development of additional chromosomal abnormalities due to segregation errors that result from having multiple centrosomes and extra chromosomes [3], [26]. The diverse chromosomal landscape of the resulting cells may then provide a permissive substrate on which selective forces can act to mould the development of a tumor. In prostate cancer, abnormal diploid cancers may represent an early stage in ploidy progression and DNA ploidy abnormalities occur in benign prostatic tissue adjacent to many prostate cancers [27], [28]. In breast cancer, there is also a correlation between aneuploidy and tumor progression [29]–[31]. However, despite the correlation between aneuploidy and tumorigenicity, direct tests for the role of aneuploidy in tumor development have been difficult partly due to the lack of suitable experimental systems, especially those consisting of human cells. One of the more direct tests of the role of tetraploidy in promoting tumorigenicity relied on chemical treatment with a cytokinesis inhibitor, dihydrocytochalasin B (DCB), to induce tetraploidy in p53-null mouse mammary epithelial cells (MMECs) [13]. In this study, the authors demonstrated that tetraploidy can promote chromosomal instability and tumorigenesis. Our data are consistent with this conclusion. However our system differs in several respects from the MMEC model: (i) We employed human epithelial cells, which are difficult to transform [32], [33]. (ii) The development of polyploidy in our system is spontaneous, following the expression of an oncogene, Pim-1. As many cancer-related genes have been linked to the development of polyploidy, for example MYC [34], APC [35], [36], Pim-1 [18], [19], BRCA2 [37], and Aurora-A [38], our model may reflect an actual pathway for tumor initiation. (iii) In our system, polyploid cells arise in the face of an intact p53 pathway, whereas p53^+/+^ tetraploid MMECs did not survive [13]. It has been reported that p53 loss facilitates the formation of tetraploidy and the survival of cells with genomic instability [39], [40].

Some studies posit the existence of a p53-dependent checkpoint that prevents the propagation of tetraploid cells [41], while other studies have questioned the existence of such a “tetraploidy checkpoint” [4], [22]. Nevertheless, tetraploid cells appear to be generally less fit than diploid cells. For example tetraploid cells are reported to have an elevated rate of spontaneous apoptosis that is dependent on p53 expression [42]. Importantly, Pim-1 has been reported to enhance cell survival through upregulation of Bcl-2 [21], as well as inactivation of the pro-apoptotic Bad protein by phosphorylation [43]. Therefore, it is possible that Pim-1, with its pro-survival functions, substitutes for p53 loss to allow for the survival of polyploid cells in our system.

Further analyses of polyploid cells before and after tumor formation are required to gain additional insights into the promotion of tumorigenesis by polyploidy. Nevertheless, it is interesting to note that the aneuploidy hypothesis predicts long latency for tumorigenesis as well as clonality. If the driving force for tumorigenesis is the inherent aneuploid karyotype initiated by a carcinogen or arising spontaneously, the resulting chromosomal instability will promote the appearance of preneoplastic and eventually neoplastic karyotypes. The generation of a neoplastic cell species will be expected to be slow and thus clonal [26], [44]. Tumorigenicity induced by polyploidy may also follow divergent routes depending on the particular karyotype generated. Consistent with this notion, the polyploid prostate and mammary epithelial cells we generated displayed very distinctive karyotypes although both cells were tumorigenic. They displayed some phenotypic differences as well. The RWPE-Pim-1 polyploid cells had a similar *in vitro* proliferation rate compared to their diploid counterparts while hTERT-HME1-Pim-1 polyploid cells proliferated at a much faster rate than the diploid cells.

In summary, our results provide evidence that polyploidy, by promoting the development of aneuploidy, is a promoter of tumorigenesis in human cells. This fact, coupled with the observation that many human tumors exhibit polyploidy, make polyploid cells attractive targets for novel therapeutics.

## Materials and Methods

### Cells, Animals, and Reagents

RWPE1 prostate and hTERT-HME1 mammary cell lines stably expressing Pim-1 were generated as described [18], [19]. Early, middle and late passage cells are defined based on the appearance of polyploidy upon in vitro culture as described previously [19]; early passage 1–20; middle passage 25–40; late passage >40. Six to eight week old athymic nude mice, male severe combined immunodeficient (SCID) mice [C.B.17/IcrHsd-scid], and pregnant Sprague-Dawley rats were obtained from NCI-Fredrick, The Jackson Laboratory or Harlan Laboratory, respectively. Antibodies used for western blotting include anti-Pim-1 (12H8), Myc (9E10), Cyclin E (M-20), Cyclin D2 (C-17), Bcl-2 (100), Bcl-_XL_ (H-5), p53 (DO-1), p21 (F-5) and actin (C-11) (all from Santa Cruz Biotechnology). Antibodies against p63 (PPM 201 AA, H, Biocare Medical), phospho-histone H3 S10 (06-570, Upstate), and Ku 70 (as10878, abcam) were used for immunostaining.

### Flow Cytometric Analysis

Cells were fixed with 70–100% cold ethanol and DNA was stained with propidium iodide for analysis as described [18], [19]. Sorting of cells based on DNA content was performed as described [19]. Briefly, intermediate passage (passage 25) cells were stained with 5 ug/ml of Hoechst 33342 (Sigma-Aldrich) for 90 min at 37°C and sorted according to DNA content (2N, 4N, and >4N) using a cell sorter. Diploid (2N DNA) and polyploid (>4N DNA) cells were used for our experiments. 4N DNA cells were not used since this is the mixed population of G2/M phase of 2N DNA cells and G1 phase of >4N DNA cells.

### Western Blotting and Immunohistochemistry

For western blot analysis, total cell lysates and samples were prepared and processed as described [18], [19] with the indicated antibodies. For daunorubicin treatment, both RWPE-1 or hTERT-HME1 cells were treated with 0.5 µm daunorubicin (Sigma-Aldrich, D8809) for 6 and 24 h, and total cell lysates were prepared for western blotting. Immunohistochemistry was carried out as previously described [45].

### Soft Agar Assays

These were carried out as described [46]. Briefly, 10^4^ cells were mixed with 0.3% soft agar and plated on top of 0.6% bottom agar seeded on each of a 60 mm plate. Triplicate plating was carried out for each sample. The cells were incubated at 37°C for 2 weeks to allow colony formation, and stained with 0.05% crystal violet for colony counting. Images were captured with a Leica DM IRB inverted wide field microscope with a Nikon DXM1200C camera.

### Xenograft and Tissue Recombination Assays

For xenografts, 3×10^6^ RWPE1 cells were mixed with 400 µl of matrigel and injected into 6–8 week old athymic nude mice subcutaneously. Mice were sacrificed after 8 months for histological analysis. Tumor size was measured with calipers and the histology of all samples was examined after sacrifice. For tissue recombination, 10^5^ sorted RWPE1-Pim-1 as well as control RWPE1-Neo cells were recombined with 2.5×10^5^ rat urogenital mesenchyme (UGM) and suspended in rat tail collagen (50 µL/graft). Rat collagen was prepared as described previously [25]. Briefly, tails from mature rats were taken and soaked in 70% ethanol, and then the skin was split at the tail root and peeled away. The tails were cut away and each tendon was teased to separate the fiber. Then tendons were transferred to acetic acid, centrifuge, and stored at 4°C until use. Rat UGM was prepared from 18-day embryonic fetuses. Urogenital sinuses were dissected from fetuses and separated into epithelial and mesenchymal components by tryptic digestion as described previously [25]. Single cells of UGM were then prepared by a 90-min digestion at 37°C with 187 units/ml collagenase (Gibco-BRL). The recombinants were incubated overnight in a 5% CO_2_ humidified incubator at 37°C in RPMI1640 and subsequently placed beneath the renal capsule of male athymic mice. Testosterone pellets were implanted dorsally under the skin of SCID mice. 12 weeks after grafting, the hosts were sacrificed. Harvested grafts were fixed in paraffin and embedded for histological and immunohistochemical analysis as described [45]. Experiments were performed according to the protocols approved by the Institutional Animal Care and Use Committees at the University of Alabama at Birmingham and at Vanderbilt University.

### Fluorescence in situ hybridization (FISH) and Spectral Karyotyping (SKY)

For FISH, cells were prepared and processed as described [14]. SKY analysis was done as described [47] and was carried out at the Roswell Park Cancer Institute SKY Analysis Facility. Briefly, the metaphase chromosomes were prepared by treatment cells with either Colcemid at 0.06 µg/ml or Nocodazole at 0.5 µg/ml for 2–4 hr. The SKY-DAPI images were captured using a Nikon microscope equipped with a Spectral cube and Interferometer module. Spectral karyotypes were prepared using SKY View software (Version 1.62). Numerical chromosomal abnormalities were determined based on the deviation from 46 (for diploid cells) or 92 (for tetraploid cells) chromosomes per cell.

## Supporting Information

Methods S1 Immunofluorescence methods for Figure S3.
(0.03 MB DOC)

Figure S1 The relationship of chromosomal abnormalities to the number of chromosomes. The number of structural chromosomal aberrations per cell or per chromosome was plotted with the data presented in Figure 3 (RWPE1 cells) and Figure 6 (hTERT-HME1 cells).
(1.04 MB TIF)

Figure S2 Late passage, polyploid, Pim-1 expressing hTERT-HME1 cells are tumorigenic in vitro. (A) Western blot for Pim-1 in early and late passage human telomerase immortalized mammary epithelial (hTERT-HME1) cells stably expressing Pim-1. (B) Cell cycle profile of Pim-1 overexpressing hTERT-HME1 cells. (C) Soft agar assay of hTERT-HME1-Pim-1 cells. (D) Soft agar colonies larger than 1 mm in diameter were counted from 60 mm dishes. The data represent the average from triplicate experiments. *p<0.05.
(1.76 MB TIF)

Figure S3 Expression of cell markers in sorted diploid and polyploid-hTERT-HME1 cells. Expression level for CD44, cytokeratin 5, and nestin were examined by immunofluorescence in diploid and polyploid hTERT-HME1 cells. There are no significant differences between these two cells. Inset, higher magnification image of nestin stain.
(2.87 MB TIF)
