# Supplementary material for: A Role for Polyploidy in the Tumorigenicity of Pim-1-Expressing Human Prostate and Mammary Epithelial Cells
Source: PLoS One. 2008 Jul 2;3(7):e2572. doi: 10.1371/journal.pone.0002572 (PMC2440349; doi:10.1371/journal.pone.0002572)
Supplement: Methods S1 — Immunofluorescence methods for Figure S3. (0.03 MB DOC) [file pone.0002572.s001.doc]

**Methods S1**

**Immunofluorescence**

Diploid and polyploid hTERT-HME1 cells were cultured on cover slides and processed for immunostaining. Briefly, cells were fixed in ice-cold methanol for 10 min, washed with PBS, and blocked with 3 % milk for 30 min at room temperature. Then, cells were incubated with anti-CD44 (sc-7297, Santa Cruz), anti-cytokeratin CK5 (C-7785, Sigma-Aldrich), or anti-nestin (sc-21248, Santa Cruz) antibodies overnight at 4°C and subsequently incubated with Alexa Fluor 488-conjugated secondary antibody (Molecular Probes) for 1 hr at room temperature. Cells were washed with PBS and mounted using Vectashield mounting medium containing 4’, 6-diamidino-2-phenylindole to counterstain nuclei (Vector Laboratories).
