# Supplementary figures and images for: A Role for Polyploidy in the Tumorigenicity of Pim-1-Expressing Human Prostate and Mammary Epithelial Cells
Source: PLoS One. 2008 Jul 2;3(7):e2572. doi: 10.1371/journal.pone.0002572 (PMC2440349; doi:10.1371/journal.pone.0002572)

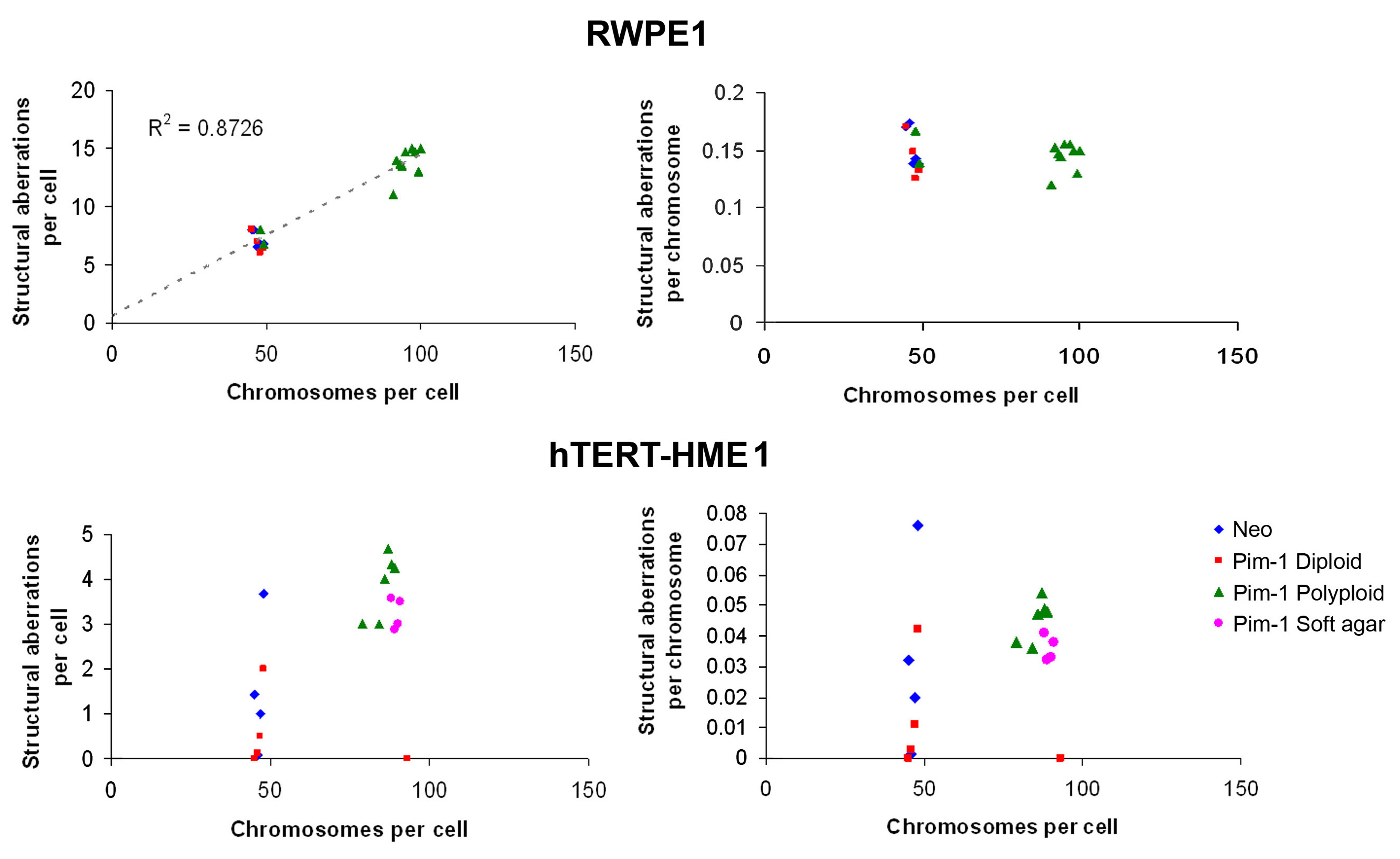

Supplement: Figure S1 — The relationship of chromosomal abnormalities to the number of chromosomes. The number of structural chromosomal aberrations per cell or per chromosome was plotted with the data presented in Figure 3 (RWPE1 cells) and Figure 6 (hTERT-HME1 cells). (1.04 MB TIF) [file pone.0002572.s002.tif]

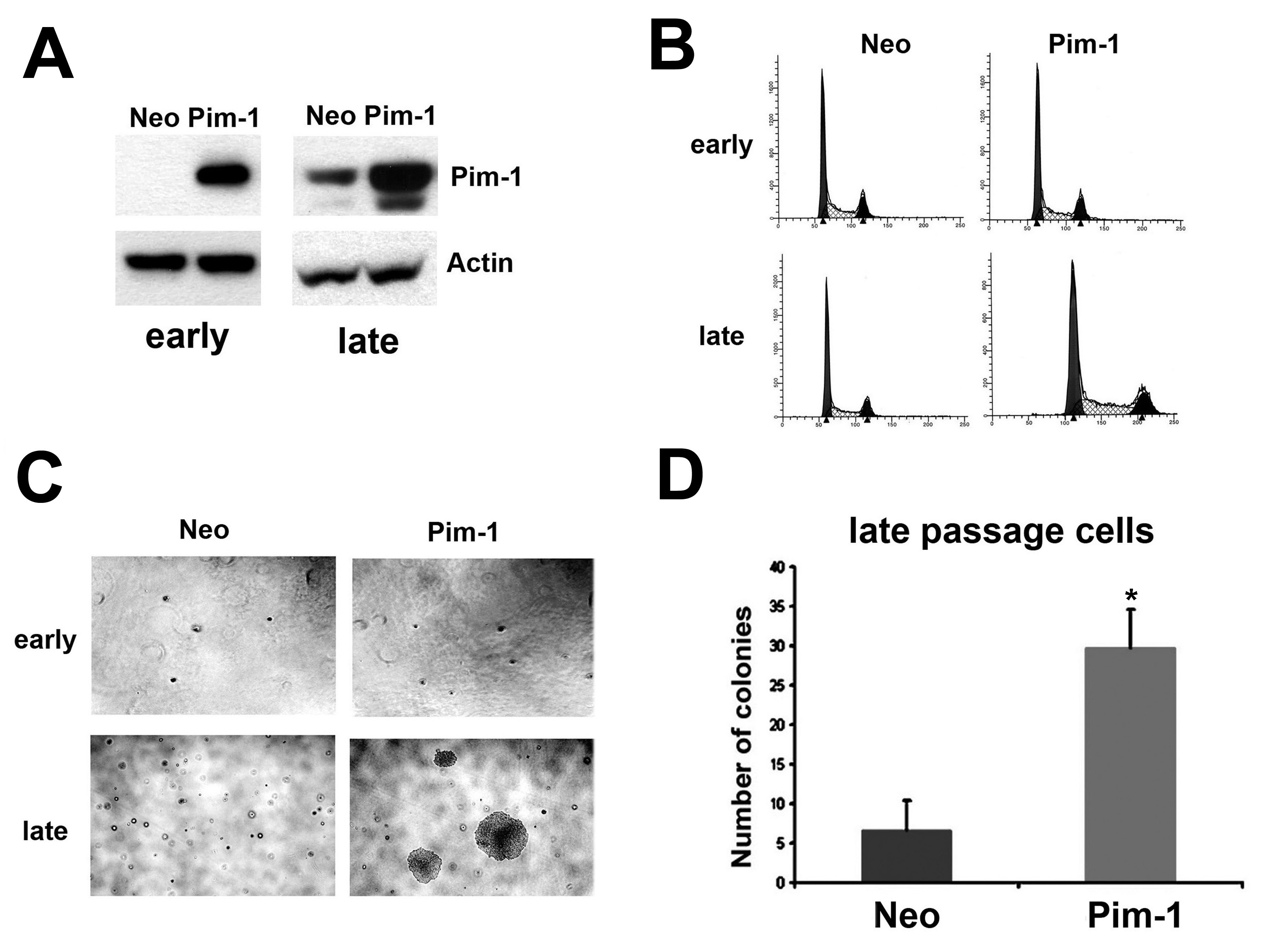

Supplement: Figure S2 — Late passage, polyploid, Pim-1 expressing hTERT-HME1 cells are tumorigenic in vitro. (A) Western blot for Pim-1 in early and late passage human telomerase immortalized mammary epithelial (hTERT-HME1) cells stably expressing Pim-1. (B) Cell cycle profile of Pim-1 overexpressing hTERT-HME1 cells. (C) Soft agar assay of hTERT-HME1-Pim-1 cells. (D) Soft agar colonies larger than 1 mm in diameter were counted from 60 mm dishes. The data represent the average from triplicate experiments. *p<0.05. (1.76 MB TIF) [file pone.0002572.s003.tif]

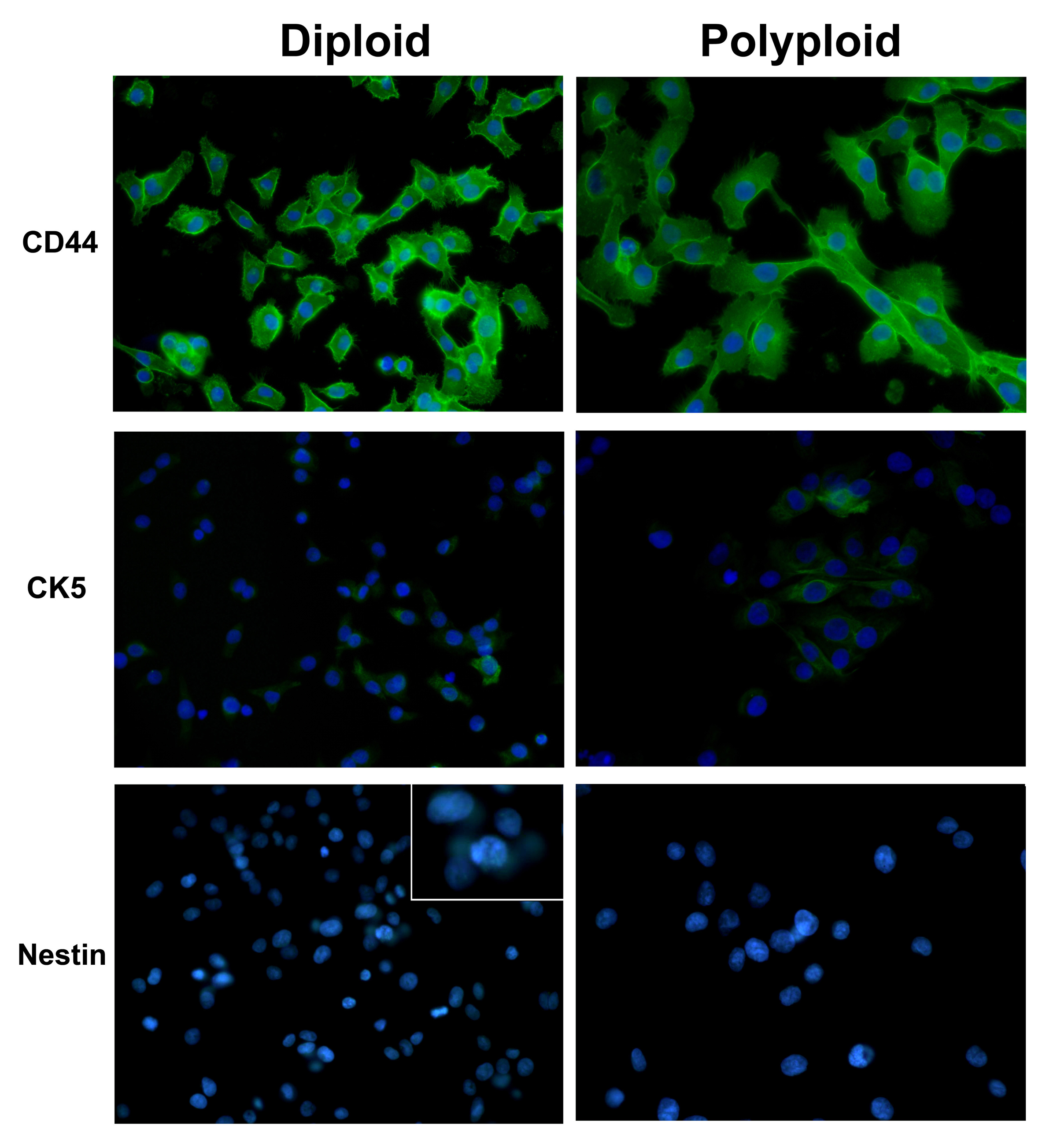

Supplement: Figure S3 — Expression of cell markers in sorted diploid and polyploid-hTERT-HME1 cells. Expression level for CD44, cytokeratin 5, and nestin were examined by immunofluorescence in diploid and polyploid hTERT-HME1 cells. There are no significant differences between these two cells. Inset, higher magnification image of nestin stain. (2.87 MB TIF) [file pone.0002572.s004.tif]
